# A Neuron-Optimized CRISPR/dCas9 Activation System for Robust and Specific Gene Regulation

**DOI:** 10.1523/ENEURO.0495-18.2019

**Published:** 2019-03-07

**Authors:** Katherine E. Savell, Svitlana V. Bach, Morgan E. Zipperly, Jasmin S. Revanna, Nicholas A. Goska, Jennifer J. Tuscher, Corey G. Duke, Faraz A. Sultan, Julia N. Burke, Derek Williams, Lara Ianov, Jeremy J. Day

**Affiliations:** 1 Department of Neurobiology and Evelyn F. McKnight Brain Institute, University of Alabama at Birmingham, Birmingham, AL, 35294; 2 Civitan International Research Center, University of Alabama at Birmingham, Birmingham, AL, 35294

**Keywords:** *Bdnf*, CRISPR, epigenetics, gene expression, transcription

## Abstract

CRISPR-based technology has provided new avenues to interrogate gene function, but difficulties in transgene expression in post-mitotic neurons has delayed incorporation of these tools in the central nervous system (CNS). Here, we demonstrate a highly efficient, neuron-optimized dual lentiviral CRISPR-based transcriptional activation (CRISPRa) system capable of robust, modular, and tunable gene induction and multiplexed gene regulation across several primary rodent neuron culture systems. CRISPRa targeting unique promoters in the complex multi-transcript gene brain-derived neurotrophic factor (*Bdnf*) revealed both transcript- and genome-level selectivity of this approach, in addition to highlighting downstream transcriptional and physiological consequences of *Bdnf* regulation. Finally, we illustrate that CRISPRa is highly efficient *in vivo*, resulting in increased protein levels of a target gene in diverse brain structures. Taken together, these results demonstrate that CRISPRa is an efficient and selective method to study gene expression programs in brain health and disease.

## Significance Statement

We report a neuron-optimized CRISPR/dCas9 activation (CRISPRa) system that produces robust and specific upregulation of targeted genes in neurons both *in vitro* and *in vivo*. This system effectively drives expression at many gene targets, provides titratable gene expression, is capable of simultaneously targeting multiple genes at once, and successfully targets individual transcript variants arising from a complex, multi-promoter gene. This molecular tool enables advances in our ability to control gene expression profiles in the brain and will enable expansion of gene regulatory investigations to model systems that have not typically been used to explore genetic control of neuronal function.

## Introduction

Gene expression patterns define neuronal phenotypes and are dynamic regulators of neuronal function in the developing and adult brain (Roth et al., 2006; Lein et al., 2007; Thompson et al., 2014). During development, differential expression of transcription factors induces gene programs responsible for neuronal fate specification and maturation (West and Greenberg, 2011). In the adult brain, specific gene programs are altered by neuronal activity and behavioral experience, and these changes are critical for adaptive behavior (Hermey et al., 2013; Benito and Barco, 2015; Duke et al., 2017). Dysregulation of both developmental and adult brain gene programs is implicated in numerous neuropsychiatric diseases, such as addiction (Robison and Nestler, 2011), depression (Jansen et al., 2016), schizophrenia (Harrison and Weinberger, 2005), and Alzheimer’s disease (Castillo et al., 2017).

Interrogating the role of gene expression programs in neuronal function has traditionally relied on the use of overexpression vectors (Prelich, 2012), transgenic animal models (Ericsson et al., 2013), and knockdown approaches such as RNA interference (Fire et al., 1998). While valuable, these techniques do not manipulate the endogenous gene locus, often require costly and time-consuming animal models, and are generally limited to one gene target at a time. Thus, while next-generation sequencing has allowed unprecedented characterization of gene expression changes in response to experience or disease, efficient multiplexed transcriptional modulation to recapitulate these expression patterns has proven elusive.

Recent advances in CRISPR/Cas9 genome editing have enabled unparalleled control of genetic sequences (Jinek et al., 2012; Straub et al., 2014; Swiech et al., 2015), transcriptional states (Konermann et al., 2015; Chavez et al., 2016), and epigenetic modifications (Savell and Day, 2017). This system has been harnessed for gene-specific transcriptional regulation by anchoring transcriptional effectors to a catalytically dead Cas9 (dCas9) enzyme, targeted to a select genomic locus with the help of a single guide RNA (sgRNA). However, these advances have not been readily adapted in the CNS due to limitations in transgene expression in post-mitotic neurons (Savell and Day, 2017). For example, reports using CRISPR-based technologies in neurons required the use of cumbersome techniques such as *in utero* electroporation (Straub et al., 2014), direct Cas9 protein infusion (Staahl et al., 2017), or biolistic transfection (Straub et al., 2014). More widespread techniques such as virus-mediated neuronal transduction have been sparsely reported for gene knockdown (Zheng et al., 2018) or activation (Frank et al., 2015; Liu et al., 2016), but the selectivity and function of these tools have not been systematically tested in neuronal systems.

Here, we present a modular, neuron-optimized CRISPR/dCas9 activation (CRISPRa) system to achieve robust upregulation of targeted genes in neurons. We show that a neuron-specific promoter is more efficient at driving the expression of CRISPR components in neurons over general ubiquitous promoters. Fusion of a robust transcriptional activator to dCas9 enabled effective gene upregulation despite gene class and size in primary rat cortical, hippocampal, and striatal neuron cultures. Co-transduction of multiple sgRNAs enabled synergistic upregulation of single genes as well as coordinated induction of multiple genes. CRISPRa targeting individual transcript promoters in brain-derived neurotrophic factor (*Bdnf*), a complex gene involved in synaptic plasticity, learning, and memory (Cunha et al., 2010), revealed highly specific *Bdnf* transcript control without impact at non-targeted variants and demonstrated the efficacy of this approach for studying downstream transcriptional programs and physiologic functions. Finally, we validated these tools for *in vivo* applications in the prefrontal cortex (PFC), hippocampus, and nucleus accumbens of the adult rat brain. Our results indicate that this neuron-optimized CRISPRa system enables specific and large-scale control of gene expression profiles within the CNS to elucidate the role of gene expression in neuronal function, behavior, and neuropsychiatric disorders.

## Materials and Methods

### Animals

All experiments were performed in accordance with the University of Alabama at Birmingham Institutional Animal Care and Use Committee. Sprague Dawley timed pregnant dams and 90- to 120-d-old male rats were purchased from Charles River Laboratories. Dams were individually housed until embryonic day (E)18 for cell culture harvest, while male rats were co-housed in pairs in plastic cages in an Association for Assessment and Accreditation of Laboratory Animal Care International-approved animal care facility on a 12/12 h light/dark cycle with *ad libitum* food and water. Animals were randomly assigned to experimental groups.

### Neuronal cell cultures

Primary rat neuronal cultures were generated from E18 rat cortical, hippocampal, or striatal tissue as described previously (Day et al., 2013; Savell et al., 2016). Briefly, cell culture plates (Denville Scientific Inc.) and microelectrode arrays (MEAs; Multichannel Systems) were coated overnight with poly-L-lysine (Sigma-Aldrich; 50 μg/ml) and rinsed with diH_2_O. Hippocampal and striatal culture plates were supplemented with 7.5 μg/ml laminin (Sigma-Aldrich). Dissected cortical, hippocampal, or striatal tissue was incubated with papain (Worthington LK003178) for 25 min at 37°C. After rinsing in complete Neurobasal media (supplemented with B27 and L-glutamine, Invitrogen), a single cell suspension was prepared by sequential trituration through large to small fire-polished Pasteur pipettes and filtered through a 100-μm cell strainer (Fisher Scientific). Cells were pelleted, re-suspended in fresh media, counted, and seeded to a density of 125,000 cells per well on 24-well culture plates (65,000 cells/cm^2^) or six-well MEA plates (325,000 cells/cm^2^). Cells were grown in complete Neurobasal media for 11 d *in vitro* (DIV) in a humidified CO_2_ (5%) incubator at 37°C with half media changes at DIV1, DIV4–DIV5, and DIV8–DIV9. MEAs received a one-half media change to BrainPhys (Stemcell Technologies Inc.) with SM1 and L-glutamine supplements starting on DIV4–DIV5 and continued every 3–4 d.

### RNA extraction and RT-qPCR

Total RNA was extracted (RNAeasy kit, QIAGEN) and reverse-transcribed (iScript cDNA Synthesis kit, Bio-Rad). cDNA was subject to RT-qPCR for genes of interest, as described previously (Savell et al., 2016). A list of PCR primer sequences is provided in Extended Data Table 1-1.

10.1523/ENEURO.0495-18.2019.t1-1 Extended Data Table 1-1 Download Table 1-1, XLSX file.

### CRISPR/dCas9 and sgRNA construct design

For transcriptional activation, a lentivirus-compatible backbone (a gift from Feng Zhang, RRID:Addgene_52961; Sanjana et al., 2014) was modified by insertion of dCas9-VPR (VP64-p65-Rta) cassette driven by one of the following promoters: human elongation factor 1α (EF1α), human phosphoglycerate kinase (PGK), CAG, and human synapsin 1 promoter (SYN). SP-dCas9-VPR was a gift from George Church (RRID:Addgene_63798; Chavez et al., 2015). For transcriptional repression, the SYN promoter was cloned into the lentivirus compatible KRAB-dCas9 construct, which was a gift from Jun Yao (Zheng et al., 2018). A guide RNA scaffold (a gift from Charles Gersbach, RRID:Addgene_47108; Perez-Pinera et al., 2013) was inserted into a lentivirus compatible backbone, and EF1α-mCherry was inserted for live-cell visualization. A *BbsI* cut site within the mCherry construct was mutated with a site-directed mutagenesis kit (NEB). Gene-specific sgRNA targets were either selected from previous studies or designed using online tools provided by the Zhang Lab at MIT (crispr.mit.edu) and CHOPCHOP (RRID:SCR_015723; http://chopchop.cbu.uib.no/; Montague et al., 2014; Labun et al., 2016). Guides were designed within –1730/+80 bp of the transcription start site (TSS) of the targeted gene as recommended previously (Mali et al., 2013; Maeder et al., 2013; Konermann et al., 2015), with most guides within the proximal promoter (∼500 bp of the TSS). To ensure specificity, all CRISPR RNA (crRNA) sequences were analyzed with National Center for Biotechnology Information’s (NCBI) Basic Local Alignment Search Tool (BLAST). A list of the target sequences is provided in Extended Data Table 1-1. Custom crRNAs were ordered as oligonucleotide sequences (Sigma Aldrich) with 5’ 4-bp overhangs (CACC for the sense strand, AAAC for the antisense strand). crRNAs were annealed, phosphorylated with PNK (NEB), and ligated using T4 ligase (NEB) into the sgRNA scaffold using the *BbsI* cut sites with unique overhangs mentioned above. For crRNA sequences that did not begin with a guanine, the first base of the crRNA sequence was substituted to guanine to maintain compatibility with the U6 promoter. Plasmids were sequence-verified with Sanger sequencing using a primer specific to the U6 promoter of the sgRNA construct. The bacterial *LacZ* gene target was used as a sgRNA non-targeting control (Platt et al., 2014).

### Transfection

HEK293T cells were obtained from American type Culture Collection (ATCC catalog #CRL-3216, RRID:CVCL_0063) and were maintained in DMEM + 10% FBS. Cells were seeded at 80 k in 24-well plates the day before transfection, and 500 ng of plasmid DNA was transfected in molar ratio (sgRNA:dCas9-VPR) with FuGene HD (Promega) for 40 h before RNA extraction and downstream RT-qPCR analysis.

### Nucleofection

C6 cells were obtained from American type Culture Collection (ATCC catalog #CCL-107, RRID:CVCL_0194) and cultured in F-12k-based medium (2.5% bovine serum, 12% horse serum). At each passage, cells were processed for nucleofection (2 × 10^6^ cells/group). Cell pellets were resuspended in nucleofection buffer (5 mM KCl, 15 mM MgCl, 15 mM HEPES, 125 mM Na_2_HPO_4_/NaH_2_PO_4_, and 25 mM mannitol) and nucleofected with 3.4-μg plasmid DNA per group. Nucleofector 2b device (Lonza) was used according to the manufacturer’s instruction (C6, high efficiency protocol). Nucleofection groups were diluted with 500-μl media and plated in triplicates in 24-well plates (∼666,667 cells/well). Plates underwent a full media change 4–6 h after nucleofection and were imaged and processed for RT-qPCR after 16 h.

### Lentivirus production

For large scale viruses, viruses were produced in a sterile environment subject to BSL-2 safety by transfecting HEK-293T cells with the specified CRISPR plasmid, the psPAX2 packaging plasmid, and the pCMV-VSV-G envelope plasmid (RRID:Addgene_12260; RRID:Addgene_8454) with FuGene HD (Promega) for 40–48 h in supplemented Ultraculture media (L-glutamine, sodium pyruvate, and sodium bicarbonate) in either a T75 or T225 culture flask. Supernatant was passed through a 0.45-μm filter and centrifuged at 25,000 rpm for 1 h 45 min at 4°C. The viral pellet was resuspended in 1/100th supernatant volume of sterile PBS and stored at –80°C. Physical viral titer was determined using Lenti-X qRT-PCR Titration kit (Takara), and only viruses >1 × 10^9^ GC/ml were used. Viruses were stored in sterile PBS at –80°C in single-use aliquots. For smaller scale virus preparation, each sgRNA plasmid was transfected in a 12-well culture plate as described above. After 40–48 h, lentiviruses were concentrated with Lenti-X concentrator (Takara), resuspended in sterile PBS, and used immediately or stored at –80°C in single use aliquots.

### Proviral integration and expression

DNA and RNA were extracted from neuronal cultures using a commercially available kit (Allprep DNA/RNA Mini with DNase treatment, QIAGEN). DNA was quantified (Quant-it dsDNA Assay kit, high sensitivity, Invitrogen) and 350 ng of genomic DNA was sonicated to 200–500 bp (Bioruptor Pico, Diagenode). Lentivirus integration (proviral DNA) was measured using qPCR with primers specific to the dCas9-VPR fusion, and normalized to *Gapdh* gDNA as a reference control. RT-qPCR was performed as outlined above to measure dCas9-VPR mRNA expression (using *Gapdh* as a reference control) for PGK, SYN, and EF1α promoters, as well as a non-transduced control.

### Immunocytochemistry and Immunohistochemistry

Immunocytochemistry (ICC) was performed as described previously (Savell et al., 2016). To validate expression of the dCas9-VPR cassette, anti-FLAG primary antibody (1:5000 in PBS with 10% Thermo Blocker BSA and 1% goat serum, Thermo Fisher Scientific catalog #MA1-91878, RRID:AB_1957945) was incubated overnight at 4°C. Cells were washed three times with PBS and incubated for 1 h at room temperature with a fluorescent secondary antibody (Alexa Fluor 488 goat anti-mouse, Thermo Fisher Scientific catalog #A-10667, RRID:AB_2534057, 1:500). Cells were washed three times with PBS and mounted onto microscope coverslips with Prolong Gold anti-fade medium (Invitrogen) containing 4,6-diamidino-2-phenylindole (DAPI) stain as a marker for cell nuclei. For immunohistochemistry (IHC), adult male rats were transcardially perfused with formalin (1:10 dilution in PBS, Fisher). Brains were removed and postfixed for 24 h in formalin, then sliced at 50 μm using a vibratome. Cells were permeabilized with 0.25% Triton X-100 in PBS, then blocked for 1 h at room temperature with blocking buffer (1× PBS with 10% Thermo Blocker BSA and 1% goat serum). To quantify the number of Fosb+ cells, slices were incubated with an anti-Fosb primary antibody (Abcam catalog #ab11959, RRID:AB_298732, 1:1000 in PBS with 10% Thermo Blocker BSA and 1% goat serum) and processed as outlined above; 20× images of each infusion site were taken on a Nikon TiS inverted fluorescent microscope by first locating the center of the mCherry signal in the targeted region and using this as a region of interest for imaging for Fosb immunoreactivity. Fosb+ cells were calculated from one projected Z stack per animal per brain region in ImageJ following background subtraction. Automated cell counts were obtained from each image using 3D object counter v2.0, with thresholds set at the same levels for both *LacZ* and *Fosb* sgRNA targeted regions within the same animal and between all animals with the same targeted region. To quantify the overlap between Fosb signal and either NeuN or GFAP, slices were incubated with an anti-Fosb antibody as described above and with an anti-NeuN (1:1000 in PBS with 10% Thermo Blocker BSA and 1% goat serum, Thermo Fisher Scientific catalog #PA5-78499, RRID:AB_2736206) or anti-GFAP (1:5000 in PBS with 10% Thermo Blocker BSA and 1% goat serum, Thermo Fisher Scientific catalog #PA1-10019, RRID:AB_1074611) and processed as outlined above with the exception of secondary antibodies used for visualization: anti-Fosb (1:500, IRDye 680RD goat anti-mouse, LI-COR Biosciences catalog #925-68070, RRID:AB_2651128) and NeuN/GFAP (Alexa Fluor 488 goat anti-rabbit, Thermo Fisher Scientific catalog #A-11034, RRID:AB_2576217, 1:500); 63× images were taken on a Zeiss LSM-800 confocal microscope by first locating the center of the mCherry signal in the targeted region, and then imaging Fosb and either NeuN or GFAP immunoreactivity. A cross-correlation analysis was performed in ImageJ with the Van Steensel’s CCF function with a pixel shift of 200 to generate the signal overlap for each of eight projected Z stack images per animal.

### Western blotting

Protein was extracted alongside RNA by collecting the flow-through from RNeasy Mini columns (QIAGEN) and precipitating protein. Each protein sample (from ∼250,000 cells) was resuspended in 25-μl RIPA lysis buffer (50 mM Tris- HCl, 150 mM NaCl, 1% NP-40, 0.5% sodium deoxycholate, 0.1% SDS and 1× Halt protease and phosphatase inhibitor; Pierce), boiled at 95°C for 5 min with 4× Laemmli buffer (Bio-Rad), separated on a 4–15% polyacrylamide gel, and transferred to a polyvinylidene difluoride membrane. BDNF protein was detected with a rabbit monoclonal anti-BDNF antibody (1:1000; Abcam catalog #ab108319, RRID:AB_10862052), and imaged on an Azure c600 imager (Azure Biosystems) using a goat anti-rabbit secondary (1:10,000; IR dye 800, LI-COR Biosciences catalog #827-08365, RRID:AB_10796098). As a loading control, β-Tubulin was detected using a mouse anti-β-Tubulin antibody (1:2000; Millipore catalog #05-661, RRID:AB_309885) and imaged using a goat anti-mouse secondary antibody (1:10,000; IR dye 680, LI-COR Biosciences catalog #926-68170, RRID:AB_10956589). Protein levels were quantified in ImageJ, and BDNF intensity values were normalized to β-Tubulin for analysis. Recombinant BDNF protein (Peprotech 450-02-10UG) was used as a positive control. For rat neuronal BDNF quantification, proBDNF (∼28 kDa) appeared as the dominant BDNF signal over mature BDNF (∼13 kDa), and was used for quantification.

### MEA recordings

Single neuron electrophysiological activity was recorded using a MEA2100 Lite recording system (Multi Channel Systems MCS GmbH). E18 rat primary hippocampal neurons were seeded in six-well MEAs at 125,000 cells/well (325,000 cells/cm^2^), as described above. Each MEA well contained nine extracellular recording electrodes and a ground electrode. Neurons were transduced with CRISPRa constructs on DIV4–DIV5 and 20-min MEA recordings were performed at DIV7, DIV9, and DIV11 while connected to a temperature-controlled headstage (monitored at 37°C) containing a 60-bit amplifier. Electrical activity was measured by an interface board at 30 kHz, digitized, and transmitted to an external PC for data acquisition and analysis in MC_Rack software (Multi Channel Systems). All data were filtered using dual 10 Hz (high pass) and 10,000 Hz (low-pass) Butterworth filters. Action potential thresholds were set manually for each electrode (typically >4 SDs from the mean signal). Neuronal waveforms collected in MC_Rack were exported to Offline Sorter (Plexon) for sorting of distinct waveforms corresponding to multiple units on one electrode channel, and confirmation of wave form isolation using principal component analysis, inter-spike intervals, and auto- or cross-correlograms. Further analysis of burst activity and firing rate was performed in NeuroExplorer. Researchers blinded to experimental conditions performed all MEA analyses.

### RNA-sequencing (RNA-Seq)

RNA-Seq was conducted at the Heflin Center for Genomic Science Genomics Core Laboratories at the University of Alabama at Birmingham. RNA was extracted, purified (RNeasy, QIAGEN), and DNase-treated for three biological replicates per experimental condition. A total of 1 μg of total RNA underwent quality control (Bioanalyzer) and was prepared for directional RNA sequencing using SureSelect Strand Specific RNA Library Prep kit (Agilent Technologies) according to manufacturer's recommendations. Poly A+ RNA libraries underwent sequencing (75-bp paired-end directional reads; ∼22–38 M reads/sample) on an Illumina sequencing platform (NextSeq2000).

### RNA-Seq data analysis

Paired-end FASTQ files were uploaded to the University of Alabama at Birmingham’s High Performance Computer cluster for custom bioinformatics analysis using a pipeline built with snakemake (Köster and Rahmann, 2018; v5.1.4). Read quality, length, and composition were assessed using FastQC before trimming low quality bases (Phred < 20) and Illumina adapters (Trim_Galore! v04.5). Splice-aware alignment to the Rn6 Ensembl genome assembly (v90) was performed with STAR (Dobin et al., 2013) v2.6.0c. An average of 88.4% of reads were uniquely mapped. Binary alignment map (BAM) files were merged and indexed with Samtools (v1.6). Gene-level counts were generated using the featureCounts (Liao et al., 2014) function in the Rsubread package (v1.26.1) in R (v3.4.1), with custom options (isGTFAnnotationFile = TRUE, useMetaFeatures = TRUE, isPairedEnd = TRUE, requireBothEndsMapped = TRUE, strandSpecific = 2, and autosort = TRUE). DESeq2 (Love et al., 2014; v 1.16.1) in R was used to perform count normalization and differential gene expression analysis with the application of Benjamini–Hochberg false discovery rate (FDR) for adjusted *p* values. Differentially expressed genes (DEGs) were designated if they passed a *p* < 0.05 adjusted *p* value cutoff and contained basemeans >50. Manhattan plots were constructed in Prism (GraphPad). Predicted off-target sgRNA hits for *Bdnf I* and *Bdnf IV* sgRNAs were identified with Cas-OFFinder, using PAM settings for SpCas9 and the Rn6 genome assembly, tolerating up to four mismatches. All hits, as well as annotated features within 2 kbp of each off-target prediction, are listed in Extended Data Tables 4-1, 4-2.

10.1523/ENEURO.0495-18.2019.t4-1 Extended Data Table 4-1 Download Table 4-1, XLSX file.

10.1523/ENEURO.0495-18.2019.t4-2 Extended Data Table 4-2 Download Table 4-2, XLSX file.

Gene ontology (GO) analysis was conducted with co-regulated genes (genes either up- or down-regulated by both *Bdnf I* and *Bdnf IV* sgRNA treatments, as compared to *LacZ* sgRNA control) using the WEB-based Gene Set Analysis Toolkit (WebGestalt; Wang et al., 2017). Overrepresentation enrichment analysis was performed using non-redundant terms in biological process, molecular function, and cellular component GO categories, using the protein-coding rat genome as a reference set. Enrichment analysis applied Benjamini–Hochberg correction for multiple comparisons and required a minimum of five genes per enriched GO term category.

### Stereotaxic surgery

Naïve adult Sprague Dawley rats were anaesthetized with 4% isoflurane and secured in a stereotaxic apparatus (Kopf Instruments). During surgical procedures, an anesthetic plane was maintained with 1–2.5% isoflurane. Under aseptic conditions, guide holes were drilled using stereotaxic coordinates [all coordinates in respect to bregma (Paxinos and Watson, 2009); CA1 dHPC: AP: –3.3 mm, ML: ±2.0 mm; NAc core: AP: +1.6 mm, ML: ±1.4 mm; mPFC: AP: +3.0 mm, ML: ±0.5 mm] to target either dorsal hippocampus CA1 region, nucleus accumbens core, or medial prefrontal cortex (mPFC). All infusions were made using a gastight 30-gauge stainless steel injection needle (Hamilton Syringes) that extended into the infusion site (from bregma: CA1: –3.1 mm, NAc core: –7.0 mm, mPFC: –4.9 mm). Bilateral lentivirus microinfusions of (1.5 μl of total volume per hemisphere) were made using a syringe pump (Harvard Apparatus) at a rate of 0.25 μl/min. Injection needles remained in place for 10 min following infusion to allow for diffusion. Rats were infused bilaterally with either 1.5 μl of total lentivirus mix comprised of 0.5 μl sgRNA and 1 μl dCas9-VPR viruses in sterile PBS. After infusions, guide holes were covered with sterile bone wax and surgical incision sites were closed with nylon sutures. Animals received buprenorphine and carprofen for pain management and topical bacitracin to prevent infection at the incision site.

### Statistical analysis

Transcriptional differences from RT-qPCR experiments were compared with either unpaired Student’s *t* tests, Mann–Whitney *U* tests, or one-way ANOVA with Dunnett’s or Tukey’s *post hoc* tests where appropriate. Fosb+ cell counts in IHC experiments were compared with a ratio paired *t* test. Statistical significance was designated at α = 0.05 for all analyses. Statistical and graphical analyses were performed with Prism software (GraphPad). Statistical assumptions (e.g., normality and homogeneity for parametric tests) were formally tested and examined via boxplots.

### Data availability

Sequencing data that support the findings of this study have been deposited in Gene Expression Omnibus (GEO) with the accession number GSE117961. All relevant data that support the findings of this study are available by request from the corresponding author. All constructs have been deposited, along with maps and sequences, in the Addgene plasmid repository (RRID:Addgene_114195; RRID:Addgene_114196; RRID:Addgene_114197; RRID:Addgene_114199).

## Results

### Optimization of CRISPRa for neuronal systems

**Figure 1.**
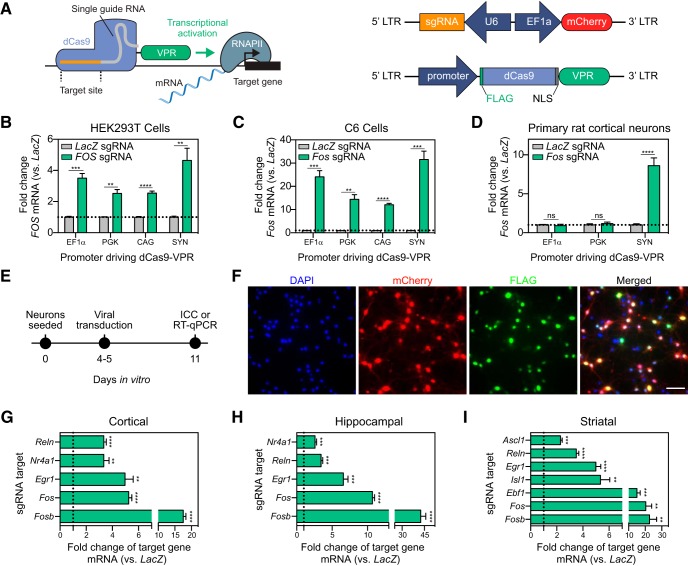
CRISPRa gene induction in HEK293T cells, C6 cells, and primary rat neurons under ubiquitous and neuron-selective promoters. ***A***, Illustration of the CRISPRa dual vector approach expressing either the sgRNA or the dCas9-VPR construct driven by EF1α, PGK, CAG, or SYN promoters. ***B***, dCas9-VPR co-transfected with sgRNAs targeted to the human *FOS* gene results in induction of *FOS* mRNA in HEK293T cells regardless of the promoter driving dCas9-VPR (*n* = 6, unpaired *t* test; EF1α *t*_(5.308)_ = 8.034, *p* = 0.0004; PGK *t*_(5.138)_ = 5.943, *p* = 0.0018; CAG *t*_(6.097)_ = 11.15, *p* < 0.0001; SYN *t*_(5.064)_ = 4.67, *p* = 0.0053). ***C***, dCas9-VPR co-nucleofected with sgRNAs targeting the rat *Fos* gene induces *Fos* mRNA in a C6 glioblastoma cell line (*n* = 6, unpaired *t* test; EF1α *t*_(5.006)_ = 8.699, *p* = 0.0003; PGK *t*_(5.067)_ = 6.640, *p* = 0.0011; CAG *t*_(5.148)_ = 18.32, *p* < 0.0001; SYN *t*_(5.000)_ = 8.631, *p* = 0.0003). ***D***, Lentiviral transduction of primary rat cortical neurons reveals that only dCas9-VPR driven by the SYN promoter results in induction of *Fos* mRNA (*n* = 6, unpaired *t* test; EF1α *t*_(6.912)_ = 0.492, *p* = 0.6378; PGK *t*_(9.491)_ = 0.710, *p* = 0.4950; SYN *t*_(5.234)_ = 7.593, *p* = 0.0005). ***E***, Experimental timeline for *in vitro* CRISPRa in neurons. Primary rat neuronal cultures are generated and transduced with dual sgRNA/dCas9-VPR lentiviruses at DIV4–DIV5. On DIV11, neurons underwent either ICC to validate viral expression or RNA extraction followed by RT-qPCR to examine gene expression. ***F***, ICC reveals high co-transduction efficiency of guide RNA (co-expressing mCherry, signal not amplified) and dCas9-VPR (FLAG-tagged) lentiviruses in primary neuronal cultures. Cell nuclei are stained with DAPI. Scale bar = 50 μm. ***G–I***, dCas9-VPR increases gene expression for a panel of genes in cortical, hippocampal, or striatal cultures. Data are expressed as fold change of the target gene’s expression relative to dCas9-VPR targeted to a non-targeting control (bacterial *LacZ* gene; *n* = 4–6, unpaired *t* test; cortical: *Reln t*_(5.438)_ = 12.590, *p* < 0.0001; *Nr4a1 t*_(3.250)_ = 5.692, *p* = 0.0086; *Egr1 t*_(5.084)_ = 6.233, *p* = 0.0015; *Fos t*_(5.571)_ = 16.770, *p* < 0.0001; *Fosb t*_(5.167)_ = 19.570, *p* < 0.0001; hippocampal: *Nr4a1 t*_(5.760)_ = 7.140, *p* = 0.0005; *Reln t*_(6.102)_ = 7.236, *p* = 0.0003; *Egr1 t*_(5.091)_ = 8.565, *p* = 0.0003; *Fos t*_(6.668)_ = 27.410, *p* < 0.0001; *Fosb t*_(5.021)_ = 12.210, *p* < 0.0001; striatal: *Ascl1 t*_(5.111)_ = 9.383, *p* = 0.0002; *Reln t*_(5.667)_ = 12.790, *p* < 0.0001; *Egr1 t*_(5.760)_ = 10.320, *p* < 0.0001; *Isl1 t*_(5.047)_ = 6.074, *p* = 0.0017; *Ebf1 t*_(5.012)_ = 7.007, *p* = 0.0009; *Fos t*_(5.026)_ = 5.349, *P* 0.003; *Fosb t*_(4.015)_ = 5.057, *p* = 0.0071). dCas9-VPR with a sgRNA targeted to the bacterial *LacZ* gene is used as a non-targeting control in panels ***B–D***, ***G–I***. All data are expressed as mean ± SEM. Individual comparisons; **p < 0.01, ***p < 0.001, ****p < 0.0001. Transgene expression and proviral integration in primary neurons are shown in Extended Data Figure 1-1. CRISPR sgRNA and RT-qPCR primer sequences are provided in Extended Table 1-1.

As highlighted by previous studies, dCas9 fusion systems containing the transcriptional activator VPR [comprised of VP64 (a concatemer of the herpes simplex viral protein VP16), p65 (a subunit of the transcription factor NF-κB), and Rta (a gammaherpesvirus transactivator)], drive expression of target genes to a much higher degree as compared to single transactivators such as VP64 or p65 alone (Gilbert et al., 2013; Mali et al., 2013; Chavez et al., 2015). To achieve high construct efficiency while balancing size constraints due to the large size of the dCas9-VPR construct (>5.5 kbp), we assembled dual lentivirus-compatible plasmid constructs (Fig. 1*A*) for separate expression of dCas9-VPR and sgRNA scaffolds. The sgRNA construct co-expresses mCherry and allows for convenient verification of expression with live cell imaging, while dCas9-VPR contains a FLAG-tag for construct expression validation through ICC (Fig. 1*A*). For dCas9-VPR cassette expression, we cloned various promoters previously shown to drive transgene expression in neurons (Yaguchi et al., 2013), including the ubiquitous promoters EF1α, PGK, and CAG (a strong synthetic hybrid promoter), as well as the neuron-specific promoter SYN. Construct functionality was validated in HEK293T cells targeting the human *FOS* gene (Fig. 1*B*). For all CRISPRa manipulations, a sgRNA targeting the bacterial *LacZ* gene paired with dCas9-VPR was used as a non-targeting control. dCas9-VPR expressed from all tested promoters successfully drove *FOS* mRNA 40 hours after transfection as measured by RT-qPCR. Before validating these constructs in rat primary neurons, we further validated rat-specific sgRNAs in C6 cells (a dividing rat glioma cell line) using nucleofection of dCas9-VPR and sgRNA plasmids targeting either *LacZ* or the rat *Fos* gene (Fig. 1*C*). Similar to HEK293T cells, dCas9-VPR expressed from all promoters was capable of inducing *Fos* mRNA. Finally, for robust expression in transfection-resistant post-mitotic neurons, we generated lentiviruses expressing sgRNA and dCas9-VPR constructs driven by various promoters. Lentiviral packaging with all dCas9-VPR plasmids generated high-titer lentiviruses (minimum 8.29 × 10^9^ GC/ml) with the exception of CAG-dCas9-VPR (likely due to exceeding recommended lentivirus capacity), which was excluded from subsequent experiments. Neuronal cultures prepared from embryonic rat cortex were transduced with either EF1α, PGK, or SYN-driven dCas9-VPR lentiviruses alongside sgRNAs targeted to either the bacterial *LacZ* or the rat *Fos* gene on DIV4, and RNA was harvested on DIV11. Surprisingly, despite transducing with the same multiplicity of infection, only the SYN-dCas9-VPR lentivirus resulted in robust induction of *Fos* mRNA (Fig. 1*D*). Taken together, our RT-qPCR results across cell lines and primary neurons indicate that while dCas9-VPR can be driven by multiple promoters in other cell types, only the SYN promoter drives sufficient transgene expression to produce a functional effect in primary neuronal cultures. To investigate the difference in promoter efficiency to drive dCas9-VPR, we measured dCas9-VPR mRNA in either EF1α, PGK, or SYN-driven dCas9-VPR transduced samples as well as a non-transduced control (Extended Data Fig. 1-1*A*). Surprisingly, the SYN-driven dCas9-VPR produced significantly more transgene mRNA compared to the other promoters despite transducing the same multiplicity of infection of each virus. It is possible that the SYN-driven virus is more efficient in proviral integration, which would explain its increased expression. To test this, we extracted genomic DNA from the same samples and measured dCas9-VPR proviral DNA using qPCR. Interestingly, we found that the PGK-driven promoter integrates more efficiently than SYN or EF1α driven dCas9-VPR (Extended Data Fig. 1-1*B*). We then normalized the mRNA expression to proviral integration and found that the SYN-driven dCas9-VPR transgene expresses dCas9-VPR to significantly higher levels as compared to PGK and EF1α promoters (Extended Data Fig. 1-1*C*). These results suggest that the SYN promoter driven dCas9-VPR construct is not more efficient at proviral integration, but is capable of expressing the transgene to a much higher level as compared to other promoters.

10.1523/ENEURO.0495-18.2019.f1-1 Extended Data Figure 1-1 dCas9-VPR transgene expression and viral integration in primary rat neurons under ubiquitous and neuron-selective promoters. ***A***, Transduction of dCas9-VPR driven by different promoters reveals that the SYN-driven transgene is more highly expressed (*n* = 6, one-way ANOVA, *F*_(3,20)_ = 12.51, *p* < 0.0001). ***B***, Transduction of dCas9-VPR driven by different promoters results in differential proviral integration with the same MOI transduced (*n* = 6, one-way ANOVA, *F*_(3,20)_ = 7.18, *p* = 0.0019). ***C***, Transduction of dCas9-VPR driven by different promoters reveals that the SYN-driven transgene is expressed to a higher degree when normalized for proviral integration (*n* = 6, one-way ANOVA, *F*_(2,15)_ = 12.69, *p* = 0.0006). All data are expressed as mean ± SEM. Tukey’s *post hoc* test for individual comparisons; *p < 0.05, **p < 0.01, ***p < 0.001. Download Figure 1-1, EPS file.

Different regions in the brain have diverse neuronal subtypes, so we next sought to validate whether the SYN-driven CRISPRa system could be used in neuronal cultures with differing neuronal composition. Primary cultures from rat embryonic cortex, hippocampus, or striatum were generated and transduced with the dual lentivirus CRISPRa system. On DIV11, cultures were used for either ICC or RNA extraction to examine gene expression with RT-qPCR (Fig. 1*E*). ICC revealed high co-localization of the sgRNA (co-expressing mCherry, signal not amplified) and the dCas9-VPR construct (FLAG-tagged) in cortical neurons (Fig. 1*F*). To assess the efficacy of the CRISPRa system at multiple gene targets, we designed one to three sgRNAs per gene targeting promoter regions 1.7 kbp upstream to 100 bp downstream of the TSS of a given target gene as previously recommended (Mali et al., 2013; Maeder et al., 2013; Konermann et al., 2015). We targeted an array of genes important to neuronal development, plasticity, and learning and memory, including immediate early genes (IEGs; *Egr1*, *Fos*, *Fosb*, *Nr4a1*), neuron-defining transcription factors (*Ascl1*, *Isl1*, *Ebf1*), and an extracellular matrix protein (*Reln*; West and Greenberg, 2011; Thompson et al., 2014; Benito and Barco, 2015). These genes varied in length from 1.8 kbp (*Ascl1*) to 426.1 kbp (*Reln*). For each targeted gene, we found significant induction of gene expression compared to the *LacZ* non-targeting control (Fig. 1*G–I*). Successful induction of a variety of targets, despite gene function or length, in multiple neuronal subpopulations suggests that this CRISPRa system can be used to drive gene expression at a large number of genes within the mammalian CNS, regardless of neuronal cell type.

### CRISPRa multiplexing enables synergistic and coordinated gene regulation

**Figure 2.**
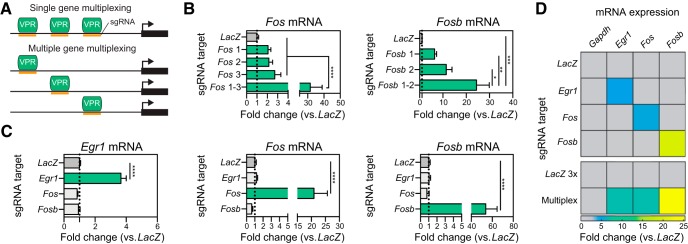
CRISPRa sgRNA multiplexing for synergistic or coordinated control of gene expression. ***A***, Illustration of pooled sgRNA multiplexing for dCas9-VPR targeting to multiple locations at a single gene (top) or simultaneous regulation of several genes (bottom). ***B***, Single gene multiplexing at *Fos* (left) and *Fosb* (right) reveals that while individual sgRNAs are sufficient to drive gene expression, sgRNA pooling results in synergistic induction of gene expression in cultured neurons (*n* = 5–6, one-way ANOVA, *Fos F*_(4,25)_ = 16.17, *p* < 0.0001; *Fosb F*_(3,19)_ = 10.23, *p* = 0.0003; Tukey’s *post hoc* test for individual comparisons). ***C***, CRISPRa with sgRNAs targeting *Egr1*, *Fos*, or *Fosb* individually results in specific and robust increases in gene expression without effects at non-targeted genes (*n* = 5–6, one-way ANOVA, *Egr1 F*_(3,16)_ = 56.53, *p* < 0.0001; *Fos F*_(3,16)_ = 17.55, *p* < 0.0001; *Fosb F*_(3,15)_ = 32.06, *p* < 0.0001; Dunnett’s *post hoc* test for individual comparisons). ***D***, Pooled gRNAs result in coordinated increases in gene expression at *Egr1*, *Fos*, and *Fosb* (*n* = 6 per group). All data are expressed as mean ± SEM. Individual comparisons; *p < 0.05, **p < 0.01, ***p < 0.001, ****p < 0.0001. CRISPR inactivation with the same sgRNAs as CRISPRa is shown in Extended Figure 2-1.

CRISPRa-mediated upregulation produced a range of magnitudes in induction between target genes. Therefore, to test whether targeting multiple copies of dCas9-VPR to a single gene boosted observed mRNA induction, we pooled between one and three sgRNA lentiviruses for each selected gene target (Fig. 2*A*). We focused on the IEGs *Fos* (three pooled sgRNAs) and *Fosb* (two pooled sgRNAs), as they produced the most robust changes in gene expression in all neuronal subpopulations. For both *Fos* and *Fosb*, combining sgRNAs synergistically induced gene expression over an individual sgRNA (Fig. 2*B*), suggesting that target gene induction can be titrated with CRISPRa to produce the desired level of gene induction.

10.1523/ENEURO.0495-18.2019.f2-1 Extended Data Figure 2-1 CRISPRi gene repression in primary striatal rat neurons employing the same sgRNAs utilized with CRISPRa. ***A***, Illustration of the CRISPRi dual vector approach expressing either the sgRNA or the KRAB-dCas9. ***B***, Lentiviral transduction of primary rat striatal neurons reveals that targeting KRAB-dCas9 to the same target sites as dCas9-VPR results in gene repression of *Egr1* and *Fosb* but not *Fos* (*n* = 6, one-way ANOVA, *Egr1 F*_(3,20)_ = 5.648, *p* = 0.0057; *Fos F*_(3,20)_ = 2.795, *p* = 0.0667; *Fosb F*_(3,20)_ = 15.120, *p* < 0.0001, Dunnett’s *post hoc* test for multiple comparisons). KRAB-dCas9 with a sgRNA targeted to the bacterial *LacZ* gene is used as a non-targeting control in panel ***B***. All data are expressed as mean ± SEM. Individual comparisons; *p < 0.05 and ***p < 0.001. Download Figure 2-1, EPS file.

Next, we sought to investigate whether the CRISPRa system could be used to drive simultaneous expression of multiple genes, providing a method to study more coordinated changes in gene expression (Fig. 2*A*). We focused on three IEGs (*Fos*, *Fosb*, *Egr1*), all of which are rapidly induced after neuronal activity and have well-established roles in neuronal function and behavior (Benito and Barco, 2015). First, we individually recruited dCas9-VPR to each gene’s promoter region in striatal cultures, which resulted in robust increases of gene expression without altering the baseline of the other genes (Fig. 2*C*). Next, we combined the sgRNA lentiviruses for all three gene targets, which resulted in simultaneous induction of all three genes (Fig. 2*D*). While we have not tested the limit of how many genes can be simultaneously induced with this system, these results demonstrate that our CRISPRa system can be used to study complex gene expression programs that normally occur in response to neuronal activation.

Previous work has introduced a CRISPR interference (CRISPRi) system in neurons, in which dCas9 is fused to a transcriptional repressor KRAB (Zheng et al., 2018). We tested whether the same sgRNAs used in our CRISPRa system could also be used to repress the same gene target with CRISPRi (Extended Data Fig. 2-1*A*). As previously described (Zheng et al., 2018), sgRNAs that are close to the TSS are most effective for transcriptional repression. We found that for *Egr1* and *Fosb*, KRAB-dCas9 targeting blunted gene expression levels (Extended Data Fig. 2-1*B*). For *Fos*, at which custom sgRNAs targeted loci at greater distances from the TSS, KRAB-dCas9 was not effective at reducing gene expression. Interestingly, we found that downregulating *Egr1* also affected baseline *Fosb* levels, suggesting that *Egr1* is necessary for *Fosb* expression. Taken together, it is possible that sgRNAs can be used for both the CRISPRa or CRISPRi systems to bidirectionally regulate gene expression.

### Selective upregulation of distinct *Bdnf* transcript variants with CRISPRa

**Figure 3.**
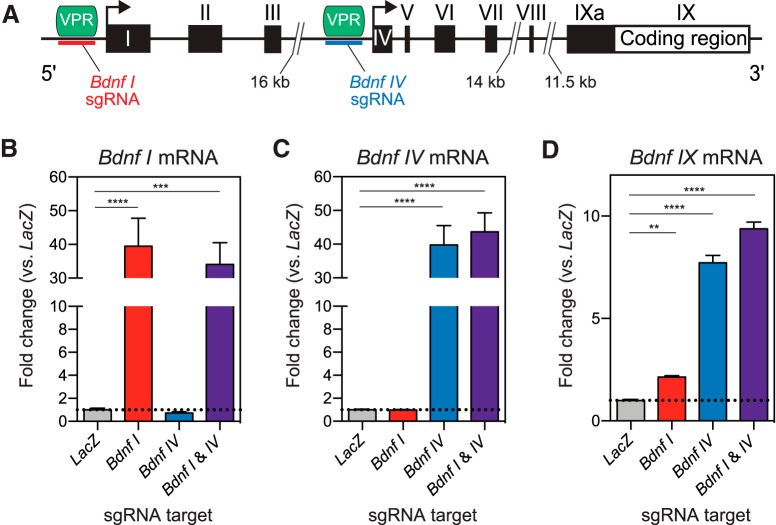
CRISPRa induction of *Bdnf* transcript variants *I* and *IV* in primary rat hippocampal neurons. ***A***, *Bdnf* gene structure illustrating non-coding exons (*I-IXa*) and a common coding exon (*IX*). sgRNAs were designed upstream of exons *I* and *IV*, as indicated by the red and blue lines. ***B–D***, Expression of *Bdnf I*, *IV*, and *IX* transcript variants after targeting dCas9-VPR to exons *I* and/or *IV* using sgRNAs, measured with RT-qPCR. ***B***, *Bdnf I* transcript is specifically upregulated with *Bdnf I* sgRNA but not with *Bdnf IV* sgRNA (*n* = 8, one-way ANOVA, *F*_(3,28)_ = 15.65, *p* < 0.0001). ***C***, *Bdnf IV* transcript is specifically upregulated with *Bdnf IV* sgRNA but not with *Bdnf I* sgRNA (*n* = 8, one-way ANOVA, *F*_(3,28)_ = 34.16, *p* < 0.0001). ***D***, Total *Bdnf IX* transcript levels are upregulated with both *Bdnf I* and *Bdnf IV* sgRNAs (*n* = 8, one-way ANOVA, *F*_(3,28)_ = 277.7, *p* < 0.0001). sgRNA designed for the bacterial *LacZ* gene is used as a non-targeting control in panels ***B–D***. Dunnett’s *post hoc* test was used for individual comparisons. All data are expressed as mean ± SEM. Individual comparisons; **p < 0.01, ***p < 0.001, ****p < 0.0001.

To examine the specificity of CRISPRa in neurons, we tested whether it is possible to drive transcription of a single transcript variant of a gene. We chose *Bdnf* as our target gene due to its complex transcriptional regulation and central role in diverse processes such as neuronal differentiation and survival, dendritic growth, synaptic development, long-term potentiation (LTP), and memory formation (An et al., 2008; Lu et al., 2008; Panja and Bramham, 2014). The *Bdnf* gene consists of nine 5’ non-coding exons (*I-IXa*) and one 3’ coding exon (*IX*; Fig. 3*A*; Aid et al., 2007). Each non-coding exon has its own unique upstream promoter region where transcription of each variant is initiated. Differential promoter usage gives rise to diverse transcripts that incorporate at least one non-coding 5’ exon in combination with the 3’ coding exon, all of which code for the same mature Bdnf protein (Aid et al., 2007). Due to this complexity, attempts to characterize distinct functional roles of individual *Bdnf* mRNAs in neurons have produced conflicting results (An et al., 2008; Baj et al., 2011), and currently available tools either lack the ability to selectively upregulate single *Bdnf* transcript variants or require cumbersome molecular cloning protocols to generate gene-specific targeting constructs.

We designed sgRNAs to target two promoter regions upstream of either *Bdnf I* or *Bdnf IV* exons. These two *Bdnf* transcripts are known to be epigenetically regulated, are responsive to neuronal stimulation, and regulate LTP and memory formation (Aid et al., 2007; Bredy et al., 2007; Lubin et al., 2008; Panja and Bramham, 2014). CRISPRa targeting at *Bdnf I* in hippocampal cultures selectively increased the expression of the *Bdnf I* transcript variant, which was also reflected in the increase of the total *Bdnf* mRNA as measured by exon *IX* upregulation (Fig. 3*B*,*D*). Likewise, co-transduction of dCas9-VPR and *Bdnf IV* sgRNA specifically upregulated the expression of *Bdnf IV* variant and also increased total *Bdnf IX* mRNA levels (Fig. 3*C*,*D*). Multiplexing both sgRNAs for *Bdnf I* and *IV* drove the expression of both transcript variants and produced a maximal upregulation of total *Bdnf IX* levels (Fig. 3*B–D*). Using *Bdnf* transcript variant manipulation, our data demonstrate specificity of the CRISPRa system at an individual mRNA transcript level.

### Transcriptome-wide selectivity of CRISPRa

**Figure 4.**
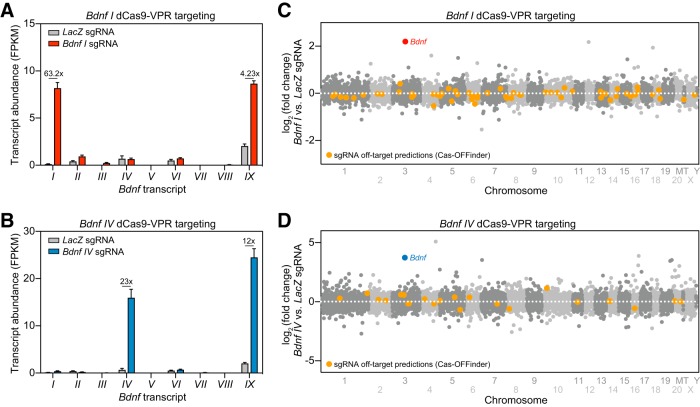
Transcriptome-wide selectivity of CRISPRa at *Bdnf* non-coding exons and the absence of off-target gene upregulation revealed by RNA-seq. ***A***, ***B***, *Bdnf* transcript variant expression (FPKM values) following dCas9-VPR targeting with *Bdnf I* (***A***) and *Bdnf IV* (***B***) sgRNAs. *Bdnf I* sgRNA treatment upregulated *Bdnf I* transcripts by 63.2x (***A***), while *Bdnf IV* sgRNA treatment upregulated *Bdnf IV* transcripts by 23x (***B***). Both *Bdnf I* and *IV* sgRNA targeted conditions increased *Bdnf IX* transcript expression by 4.23x and 12x, respectively. sgRNA designed for the bacterial *LacZ* gene is used as a non-targeting control. All data are expressed as mean ± SEM in ***A***, ***B***. ***C***, ***D***, Mirrored Manhattan plots showing degree of mRNA change across the genome for *Bdnf I* (***C***) and *Bdnf IV* (***D***) dCas9-VPR targeting. While there were no exact matches for *Bdnf I* or *Bdnf IV* sgRNA sequences elsewhere in the genome, all potential off-target sites with up to 4 nucleotide mismatches (identified with Cas-OFFinder) are shown in orange. Predicted off-target sequences for *Bdnf I* and *IV* targeting are shown in Extended Tables 4-1 and 4-2, respectively.

CRISPR-based targeting relies on complementary sequence identity between the sgRNA and genomic DNA. Therefore, off-target sgRNA binding and gene induction is possible if there is sufficient sequence similarity (Sternberg et al., 2014). To evaluate specificity with *Bdnf* transcript induction, we performed whole-transcriptome RNA-seq after CRISPRa targeting of *Bdnf I* or *IV* in hippocampal cell cultures. Quantification of transcript abundance [using fragments per kilobase per million mapped reads (FPKM) values] for each non-coding *Bdnf* exon (*I–VIII*) and the common-coding exon *IX* revealed that targeting either exon *I* or *IV* specifically increased the targeted transcript variant without altering adjacent transcripts. Targeting either exon *I* or *IV* also increased the abundance of the coding *Bdnf IX* exon (Fig. 4*A*,*B*). Although *Bdnf I* or *Bdnf IV* sgRNA sequences were completely unique within the rat genome assembly (with no complete matches elsewhere), it was possible that CRISPRa could induce off-target effects at other genes. To examine this, we performed an extensive algorithmic search for potential off-target DNA sequences using Cas-OFFinder (Bae et al., 2014), allowing systematic identification of similar sequences within the rat Rn6 genome with up to 4 nucleotide mismatches to our sgRNAs (for complete list, see Extended Data Tables 4-1, 4-2). Most potential off-target loci fell within intergenic regions distant from any annotated genes. However, even for predicted off-target sites located within or near genes (± 2 kbp), we detected few gene expression changes with either sgRNA manipulation. For *Bdnf I* CRISPRa targeting, we identified 61 predicted off-target genes (annotated in orange in Fig. 4*C*), but only 7 (11.5%) were significantly altered as compared to the *LacZ* control group (four upregulated genes and three downregulated genes). Likewise, for *Bdnf IV* sgRNA targeting, we identified 23 predicted off-target genes (Fig. 4*D*), only 6 (26.1%) of which were differentially expressed genes (DEGs; three upregulated genes and three downregulated genes vs. *LacZ* controls. Given that the percentages of predicted off-target genes significantly altered in each case were similar to the overall percentage of genes altered in *Bdnf I* and *Bdnf IV* CRISPRa targeting (5.3% and 22.9%, respectively), and that observed changes included both increases and decreases in gene expression, we interpret these results to indicate a lack of direct off-target effects using CRISPRa. Finally, genes directly upstream and downstream of *Bdnf* on the third chromosome (*Lin7c* and *Kif18a*) were not differentially expressed following either manipulation, suggesting that on-target effects do not alter the expression of nearby genes. Together, these results illustrate the selectivity of the CRISPRa system, which robustly upregulated the expression of select transcript variants of *Bdnf* without driving adjacent genes or predicted off-target loci.

### Downstream transcriptional outcomes following CRISPRa at *Bdnf*

**Figure 5.**
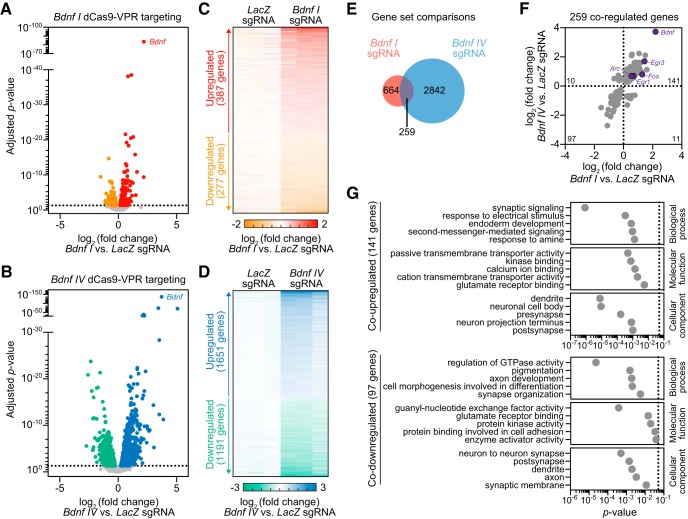
CRISPRa targeted induction of *Bdnf I* and *IV* transcript variants causes coordinated upregulation of genes involved in neuronal activation and synaptic function. ***A***, ***B***, RNA-seq volcano plots showing DEGs detected by DESeq2 in *LacZ* versus *Bdnf I* sgRNA (***A***) and *LacZ* versus *Bdnf IV* sgRNA (***B***) targeted conditions. Standard cutoff point is represented by the horizontal dotted line (adjusted *p* < 0.05). Upregulated (red or blue) and downregulated (orange or green) genes are indicated for each comparison. *Bdnf* is the top upregulated gene in both conditions. ***C***, ***D***, Heat maps representing all DEGs comparing *LacZ* versus *Bdnf I* sgRNA (***C***) and *LacZ* versus *Bdnf IV* sgRNA (***D***) targeted conditions for three biological replicates. Values in each row represent *LacZ*-normalized counts for each DEG (adjusted *p* < 0.05). Log_2_ fold change increases (red or blue) or decreases (orange or green) in gene expression are presented relative to the *LacZ* mean (white). ***E***, Venn diagram representing 664 DEGs after *Bdnf I* sgRNA targeting (red) and 2842 DEGs after *Bdnf IV* sgRNA targeting (blue), with 259 overlapping genes. ***F***, Scatter plot representing all shared 259 DEGs in *Bdnf I* versus *Bdnf IV* sgRNA targeted conditions. Genes upregulated in both groups (141), downregulated in both groups (97), upregulated after *Bdnf I* and downregulated after *Bdnf IV* sgRNA targeting (11), downregulated after *Bdnf I* and upregulated after *Bdnf IV* sgRNA targeting (10) are indicated. Select upregulated IEGs are specified. ***G***, Top significant GO terms for 141 co-upregulated and 97 co-downregulated genes in *Bdnf I* and *Bdnf IV* sgRNA targeted conditions.

To investigate the identity of genes differentially regulated by *Bdnf I* or *IV* upregulation using CRISPRa, we first characterized DEGs in either *Bdnf I* or *IV* versus *LacZ* targeted conditions. In both datasets, *Bdnf* was the top significantly upregulated gene (Fig. 5*A*,*B*). We detected 387 upregulated genes and 277 downregulated genes after *Bdnf I* induction as well as 1651 upregulated genes and 1191 downregulated genes after *Bdnf IV* targeting (Fig. 5*C*,*D*). Out of the 664 DEGs altered by *Bdnf I* upregulation and 2842 DEGs altered by *Bdnf IV* upregulation, 259 genes were shared in both conditions (Fig. 5*E*). At these 259 co-regulated genes, nearly all (238 of 259, 91.9%) were regulated in the same direction by *Bdnf I* and *Bdnf IV* targeting. Increased *Bdnf* levels were associated with elevated expression of several IEGs that are often used as markers for neuronal activation, including *Arc*, *Fos*, *Egr1*, and *Egr3* (Fig. 5*F*). These results complement previous studies linking Bdnf signaling with IEG expression (Bramham and Messaoudi, 2005; Cortés-Mendoza et al., 2013), but extend this by offering the first insights into differential gene expression regulation by unique *Bdnf* transcript variants.

GO analysis revealed co-upregulated genes shared by both *Bdnf I* and *IV*-targeting conditions were enriched for synaptic signaling, response to stimulation, and second-messenger signaling activation (Fig. 5*G*, top panel). Additionally, co-upregulated genes are enriched in molecular functions ranging from transmembrane transporter activity to kinase and glutamate receptor binding and are enriched for synaptic and projection-specific compartmentalization (Fig. 5*G*, top panel). Genes that were co-downregulated are involved in the regulation of signaling molecule activity, cell differentiation, and axonal development processes (Fig. 5*G*, bottom panel). Overall, the transcriptome-wide characterization of *Bdnf*-induced DEGs supports the role of *Bdnf* function in synaptic plasticity, neuronal signaling, response to glutamate, and activation of second-messenger systems (Bramham and Messaoudi, 2005; Panja and Bramham, 2014). This further highlights how CRISPRa can be used to drive gene expression profile changes to explore downstream molecular consequences of altered neuronal signaling.

### Physiologic alterations following CRISPRa-mediated *Bdnf* and *Reln* upregulation

**Figure 6.**
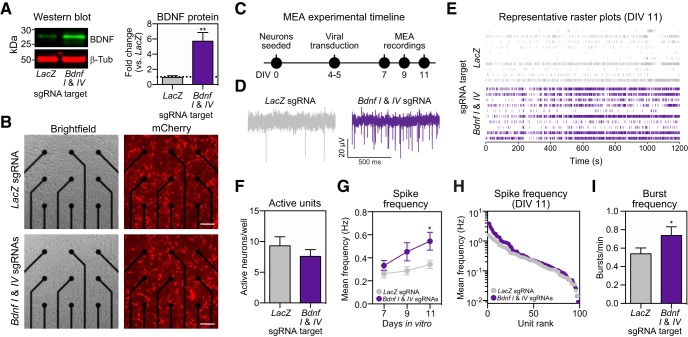
CRISPRa induction of *Bdnf* mRNA increases spike and burst frequency in hippocampal neurons cultured on microelectrode arrays (MEAs). ***A***, CRISPRa induction of *Bdnf I* and *IV* increases Bdnf protein quantified by immunoblotting (*n* = 6 per group; Mann–Whitney *U* test, *U* = 0, *p* = 0.0022). ***B***, Primary hippocampal neurons grown on MEAs and transduced with dCas9-VPR and *LacZ* (top) or *Bdnf I* and *IV* (bottom) sgRNAs. mCherry signal indicates successful transduction of sgRNAs in live cultures (right). Scale bar = 100 μm. ***C***, Experimental timeline for viral transduction and MEA recordings. Representative traces (***D***) and raster plots (***E***) from 10 units after *LacZ* (top) or *Bdnf I* and *IV* (bottom) targeting. ***F***, The number of active units per well does not change between *LacZ* and *Bdnf I* and *IV* targeted conditions (*n* = 10–12, unpaired Student’s *t* test; *p* = 0.1783). ***G***, Action potential frequency across DIV7–DIV11 showing an increase of mean frequency after *Bdnf I* and *IV* sgRNA treatment by DIV11, as compared to *LacZ* sgRNA (*n* = 57–98 neurons, two-way ANOVA with main effect of sgRNA, *F*_(1,493)_ = 8.561, *p* = 0.0036, Sidak’s *post hoc* test for multiple comparison). ***H***, Spike frequency at DIV11 for all units ranked from highest to lowest mean frequency showing an increase in activity for the top 1/3 most active units in *Bdnf I* and *IV* versus *LacZ* targeted conditions. ***I***, Burst frequency at DIV11 is increased after *Bdnf I* and *IV* versus *LacZ* targeting (*n* = 98, unpaired Student’s *t* test; *p* = 0.0392). All data are expressed as mean ± SEM; *p < 0.05 and **p < 0.01. The physiological consequences of CRISPRa induction at another gene target (*Reln*) is shown in Extended Figure 6-1.

It is well established that BDNF signaling enhances synaptic communication and facilitates the induction of LTP (Poo, 2001; Bramham and Messaoudi, 2005; Panja and Bramham, 2014). Application of exogenous BDNF protein has also been shown to enhance neuronal firing rates via regulation of intrinsic neuronal excitability and homeostatic plasticity in neuronal cultures (Desai et al., 1999) and hippocampal brain slices (Graves et al., 2016), or via depressive effects at inhibitory interneurons (Nieto-Gonzalez and Jensen, 2013). Given that our RNA-seq results indicated induction of *Bdnf* with CRISPRa increases expression of genes commonly linked to neuronal activation, we next tested whether *Bdnf* upregulation using CRISPRa influences physiologic properties of neuronal cultures. We first investigated whether induction of *Bdnf* mRNA following CRISPRa targeting to *Bdnf* I and IV promoters resulted in increased BDNF protein levels (Fig. 6*A*). Using Western blotting with an anti-BDNF antibody, we found a robust (∼6-fold) increase in BDNF protein levels following CRISPRa manipulation, but no changes in the loading control protein β-Tubulin. To investigate the physiologic properties of this manipulation, primary hippocampal neurons were seeded directly on MEAs in cell culture plates and transduced with lentiviruses expressing sgRNAs (*Lac*Z control or *Bdnf I* and *IV*) and CRISPRa machinery (Fig. 6*B*,*C*). Following neuronal transduction on DIV4, we verified expression of sgRNA lentiviral vectors using mCherry expression and performed electrophysiological recordings on DIV7, DIV9, and DIV11 (Fig. 6*C*,*D*). Compared to the non-targeting control (*LacZ* sgRNA), treatment with *Bdnf I* and *IV* sgRNAs increased action potential frequency by DIV11 without changing the number of active units across the two conditions (Fig. 6*F*,*G*). A detailed analysis of all active units ranked from highest to lowest mean frequency revealed that the increase in firing rate occurred primarily in the top one-third most active neurons (Fig. 6*H*). In addition, the frequency of action potential bursts was increased, indicating increased communication between neurons and a greater potential for enhanced synaptic plasticity (Fig. 6*I*). Collectively, these experiments demonstrate that upregulation of *Bdnf* gene expression using CRISPRa increases baseline neuronal activity patterns, which is consistent with previous reports demonstrating elevated neuronal excitability in pyramidal neurons of the hippocampus following application of recombinant BDNF protein (Desai et al., 1999; Nieto-Gonzalez and Jensen, 2013; Graves et al., 2016).

10.1523/ENEURO.0495-18.2019.f6-1 Extended Data Figure 6-1 CRISPRa targeting of *Reln* in hippocampal neurons. ***A–C***, *Reln* targeting with CRISPRa results in more active neurons at DIV7, but no change in spike or burst frequency (*n* = 15 wells, unpaired Student’s *t* test; active units *t*_(28)_ = 2.574, *p* = 0.0156). MEA recordings occurred on DIV7, approximately 72 h after viral transduction. All data are expressed as mean ± SEM. Individual comparisons; *p < 0.05. Download Figure 6-1, EPS file.

To extend these observations to a second gene, we investigated neuronal activity patterns after CRISPRa-mediated upregulation of the *Reln* gene, which codes for REELIN, a large and multifunctional extracellular protein. Bidirectional modulation of *Reln* expression has been shown to affect neuronal function and synaptic activity by altering the NDMA receptor (Chameau et al., 2009; Rogers et al., 2011). Additionally, the *Reln* locus is large, taking up ∼426 kbp of genomic DNA, making it a difficult target for traditional genetic manipulations such as cDNA overexpression cassettes. In cultured hippocampal neurons plated on MEAs and recorded on DIV7, we found that wells containing the *Reln*-targeted dCas9-VPR construct were not functionally distinct from controls in that there was not a significant difference in action potential frequency or bursting activity (Extended Data Fig. 6-1*A–C*). However, unlike *Bdnf* manipulation, upregulation of *Reln* increased the number of spontaneously active neurons. Overall, these findings suggest that CRISPRa targeting to *Reln* has dissociable effects from *Bdnf* manipulations on neuronal physiology and highlight the utility of CRISPRa approaches for investigation of genetic regulation of neuronal communication patterns.

### CRISPRa gene targeting results in increased protein levels *in vivo*

**Figure 7.**
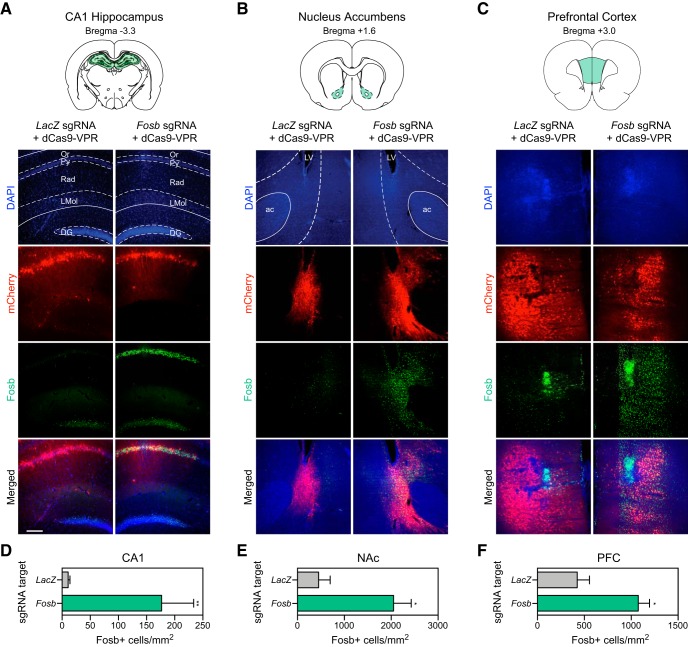
CRISPRa-mediated induction of Fosb in hippocampal, striatal, and cortical neurons *in vivo*. ***A–C***, Lentiviral infusions were bilaterally targeted to the brain region of interest (Paxinos and Watson, 2009) in adult male rats (*n* = 4 rats/region). Two weeks following stereotaxic viral infusions, animals were transcardially perfused and IHC was performed to measure Fosb upregulation. IHC reveals high transduction efficiency of the guide RNA (expressing mCherry, signal not amplified) bilaterally in (***A***) the CA1 region of the dorsal hippocampus, (***B***) the nucleus accumbens core (NAc), and (***C***) the medial PFC. Fosb protein is enhanced in the hemisphere that was infused with the *Fosb*-targeting sgRNA (right) compared to the hemisphere that received a sgRNA targeting the bacterial *LacZ* gene (left). Cell nuclei were stained with DAPI. Scale bar = 500 μm. Schematics of target regions are adapted from Paxinos and Watson. ***D–F***, dCas9-VPR increases the number of Fosb+ cells in the CA1, NAc, and PFC, compared to a non-targeting control (*LacZ*; *n* = 4, ratio paired *t* test; CA1: *t*_(3)_ = 8.73, *p* = 0.003, *R* ^2^ *=* 0.96; NAc: *t*_(3)_ = 4.62, *p* = 0.019, *R* ^2^ = 0.87; PFC: *t*_(3)_ = 3.43, *p* = 0.041, *R* ^2^ = 0.79). All data are expressed as mean ± SEM. Individual comparisons; *p < 0.05 and **p < 0.01. Or: oriens layer, Py: pyramidal cell layer, Rad: radiatum layer, LMol: lacunosum moleculare, DG: dentate gyrus, ac: anterior commissure, LV: lateral ventricle.

To examine the efficiency of the CRISPRa system *in vivo,* we stereotaxically infused CRISPRa lentivirus and sgRNA lentiviruses (non-targeting *LacZ* control or rat *Fosb*) into opposite hemispheres of the dorsal hippocampus, nucleus accumbens, or PFC of adult rats (Fig. 7*A–C*). After two weeks to allow for viral expression, animals were perfused and IHC was performed for FOSB to determine if CRISPRa targeting results in increases in protein levels. Since the mCherry signal survives fixation and does not need to be amplified with an antibody in IHC, we were able to observe the viral spread in all targeted brain regions, noting that there was robust expression of the sgRNA construct in each region regardless of *LacZ* or *Fosb* targeting. Importantly, FOSB protein expression was strongly increased only in hemispheres receiving *Fosb* sgRNAs paired with dCas9-VPR (Fig. 7*A–C*, *LacZ* targeting left, *Fosb* targeting right), indicating that increases in gene expression directly result in an increased number in FOSB+ cells in all regions (Fig. 7*D–F*). These results offer evidence that CRISPRa can be used successfully *in vivo* in multiple neuronal populations to achieve increases in protein translation with a single viral infusion of pooled dCas9-VPR and sgRNA lentiviruses in the adult brain.

### CRISPRa increases in protein levels is neuron selective

**Figure 8.**
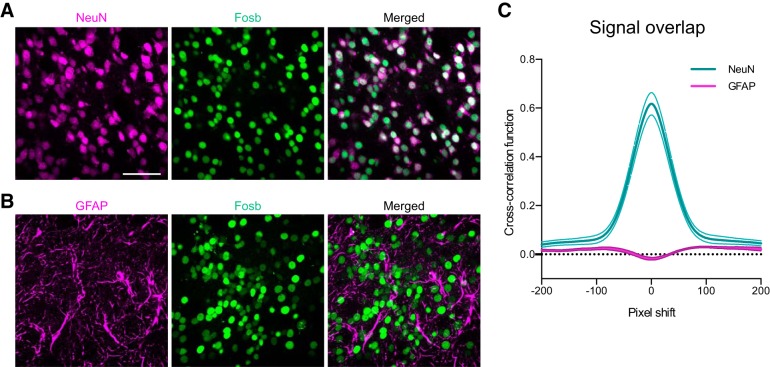
CRISPRa-mediated induction of Fosb is neuron-selective *in vivo*. ***A***, ***B***, IHC performed for (***A***) NeuN or (***B***) GFAP alongside Fosb demonstrates neuronal selectivity of CRISPRa-mediated Fosb induction. Scale bar = 50 μm. ***C***, Pixel density quantification and cross-correlation analysis reveals a signal overlap between Fosb and NeuN and depletion of signal between Fosb and GFAP (*n* = 2 animals with eight regions of interest). All data are expressed as mean ± SEM.

The dCas9-VPR construct is driven by the SYN promoter, which has previously been found to be neuron-specific *in vivo* (Jackson et al., 2016). To validate that our CRISPRa-mediated increases in FOSB protein occur in neurons but not other cell types (e.g., glia), we performed dual IHC for either NeuN (Fig. 8*A*) or GFAP (Fig. 8*B*) alongside FOSB. We observed a strong overlap between the FOSB and NeuN signal, and a depletion in the overlap between FOSB and GFAP (Fig. 8*C*). Taken together, these results suggest that protein increases generated by CRISPRa are neuron-selective *in vivo*.

## Discussion

Unraveling transcriptional control of specific neuronal properties and functions requires tools that can achieve robust, selective, and modular induction of gene expression. Here, we present a neuron-optimized CRISPRa system capable of inducing targeted endogenous gene expression in post-mitotic neurons. This system allows efficient targeting of a wide variety of genes that are critical for neuronal processes, including genes of various lengths, cellular roles, and physiologic functions. We demonstrate that this optimized CRISPRa system is effective in multiple neuronal populations, including cortical, hippocampal, and striatal neurons both *in vitro* and *in vivo.* Moreover, multiplexed pooling of sgRNAs enables synergistic upregulation of a single target or coordinated control over many genes. We highlight the unprecedented selectivity of the CRISPRa system by driving the expression of individual *Bdnf* mRNA transcript variants without globally affecting non-targeted variants or off-target genes, as well as the utility of this system for studying how single-gene manipulations alter gene expression programs and neuronal physiology. Together, these results provide compelling support for application of CRISPRa approaches to the study of gene regulation in diverse neuronal systems.

A key limitation to current gene overexpression approaches is the inability to express long genes using common viral vectors such as AAVs or lentiviruses. Our neuron-optimized lentivirus-based CRISPRa system provides an opportunity to expand the number of possible genetic screens in the CNS, especially for genes that are too long to be packaged in an overexpression vector. In this study, we successfully targeted genes of variable lengths: shorter genes such as *Ascl1* (1.8 kbp) and *Fos* (2.8 kbp), medium-length genes such as *Bdnf* (50 kbp), and longer genes such as *Reln* (426 kbp) and *Ebf1* (389 kbp). Previous studies have relied on direct recombinant protein infusion for longer genes such as Reln (Rogers et al., 2011), whose cDNA exceeds common virus vector capacities. While typical overexpression systems would require increased viral capacity to express long genes, this CRISPRa system has a fixed cargo size given that sgRNA length does not need to increase with gene size. Importantly, this lentiviral-mediated construct delivery system allows for transgene expression within one week *in vitro* and two weeks *in vivo* (Figs. 1, 7), while also providing stable genome integration for potentially long-lasting upregulation. Additionally, the greater packaging capacity of the lentiviral capsid (∼10 kbp) is ideal for the larger dCas9-VPR construct, as opposed to other viral vectors with lower packaging capacity, such as an AAV (∼4.7 kbp; Lentz et al., 2012). Moreover, these lentivirus-compatible constructs can be packaged into high-titer lentiviruses capable of high neuronal efficiency. Thus, this system can be used to drive a variety of genes regardless of length or complexity in post-mitotic neurons.

While the emergence of next-generation sequencing has allowed for unprecedented insight into the genome-wide changes in gene expression during development or in response to environmental stimuli, methods to mimic larger-scale gene expression profiles have been lacking. With CRISPRa, simultaneous activation of multiple gene targets allows for the investigation of global transcriptomic states, in addition to candidate gene approaches. At the *Fos* and *Fosb* genes, we found that pooling multiple sgRNAs drove more robust increases in gene expression, potentially enabling gene expression changes to be carefully and stably titrated to achieve alterations that mimic physiologic conditions. Likewise, we found that multiplexing sgRNAs across genes enabled simultaneous expression of genes that are often co-regulated by neuronal depolarization, enabling more effective experimental dissection of cooperative gene programs that link neuronal activation to long-term adaptive changes.

Despite using the same dCas9-VPR fusion as a transcriptional activator at all genes, we found remarkable variability in levels of gene induction following CRISPRa. This variability is likely influenced by multiple factors, including sgRNA placement relative to gene regulatory elements, chromatin accessibility, and baseline gene expression levels (Konermann et al., 2015; Chavez et al., 2016; Zhou et al., 2018). In combination with rapidly growing transcriptome- and genome-wide datasets from distinct neuronal structures and subtypes, it is likely that these factors can be effectively harnessed to establish predictable rules for gene induction across neuronal systems. Similarly, we anticipate that this approach can easily be expanded to incorporate other fusion proteins, such as gene repressors or enzymes that catalyze or remove histone and DNA modifications. Indeed, using a previously neuron-optimized CRISPRi system, we also found that some sgRNAs can be repurposed for bidirectional modulation of gene expression, demonstrating the flexibility and modular nature of this approach.

The CRISPRa system allows for the investigation of unique biological questions not feasible to study using other approaches. For example, the functional significance of exon-specific promoter usage during transcription of *Bdnf* has been a long-standing question in the field of neuroscience (Cunha et al., 2010). Differential expression of diverse *Bdnf* transcript variants have been described in numerous physiologic states, such as development and adult synaptic plasticity, as well as neurodevelopmental and psychiatric disorders such as addiction, schizophrenia, and depression (Autry and Monteggia, 2012). Here, we demonstrate exquisite selectivity of CRISPRa at a single transcript variant of *Bdnf* while leaving non-targeted *Bdnf* transcripts and potential off-target genes unaffected. RNA-seq analysis after specific *Bdnf* variant upregulation showed an enhancement of genes involved in synaptic plasticity, neuronal excitability and dendritic arborization, all consistent with the known roles of *Bdnf* in the nervous system (Panja and Bramham, 2014). Although many DEGs after *Bdnf I* or *Bdnf IV* transcript variant upregulation were shared by both conditions, some DEGs were uniquely associated with each transcript, supporting the idea that individual variants have differential functions. We cannot rule out that the differences observed in gene expression arose due to the differential magnitudes of induction of total *Bdnf*, but future studies are now poised to investigate these questions more thoroughly to elucidate the role of specific activity-dependent transcript variants. Our CRISPRa platform also yielded the novel discovery that upregulation of specific *Bdnf* variants is sufficient to elevate BDNF protein levels, leading to an increase in spike and burst frequency in cultured hippocampal neurons. While these results support previous reports that BDNF can potentiate synaptic plasticity and modulate intrinsic neuronal excitability (Desai et al., 1999; Lu et al., 2008; Cunha et al., 2010; Panja and Bramham, 2014; Graves et al., 2016), they highlight how CRISPRa could be used to investigate the function of not only individual genes, but also diverse transcript variants of genes in complex neuronal systems. Additionally, these results also provide novel evidence for a role of specific *Bdnf* transcript variants in neuronal function and downstream transcriptional regulation.

An additional advantage of this CRISPRa approach is the ease of transfer across model systems. In our studies, we used the outbred Sprague Dawley rat strain for all neuronal experiments. While this organism is commonly used to model complex behavioral and cognitive processes and is often viewed to have more relevance as a model of human disease (Ellenbroek and Youn, 2016), it has not been as readily amenable to genetic manipulations as *Drosophila melanogaster*, *Caenorhabditis elegans*, or mouse model systems. This drawback has led to generation of fewer transgenic rat lines, which delays incorporation of this important model system into investigations targeting molecular mechanisms. This newly-optimized CRISPRa system provides more avenues for mechanistic work in rats and other model species.

This CRISPRa system is comprised of a constitutively active construct. Adaptation of these CRISPRa tools for inducible systems or viral approaches that allow more transient expression will enable further flexibility of use and precise temporal control of gene expression. For example, during development, temporal regulation of gene expression is critical to establish cell phenotype and connectivity in the developing brain. In adulthood, neuronal activity alters cellular signaling cascades, which often converge in the nucleus to alter gene expression as a result of environmental stimulation. To gain even tighter temporal control on transcription, this system could be adapted into existing chemical or physical inducible systems (Savell and Day, 2017). Additionally, while this study did not target specific neuronal subpopulations with subpopulation-associated promoters (excitatory, inhibitory, and modulatory neuron-associated promoters), this addition could enable powerful circuit-specific targeting through use of cell-type specific promoters or transgenic animals expressing Cre recombinase in specific cell populations.

In short, here we establish a robust and neuron-optimized CRISPR/dCas9 activator system for specific upregulation of gene expression. The CRISPRa system is fast, inexpensive, modular, and drives potent and titratable gene expression changes from the endogenous gene loci *in vivo* and *in vitro*, making it more advantageous over traditional genetic manipulations, such as the use of transgenic animals or overexpression vectors. We propose that the CRISPRa system will be a readily accessible tool for the use in the investigation of gene function in the CNS.

## Synthesis

Reviewing Editor: Chay Kuo, Duke University Decisions are customarily a result of the Reviewing Editor and the peer reviewers coming together and discussing their recommendations until a consensus is reached. When revisions are invited, a fact-based synthesis statement explaining their decision and outlining what is needed to prepare a revision will be listed below. The following reviewer(s) agreed to reveal their identity: Jin-Wu Tsai. Note: If this manuscript was transferred from JNeurosci and a decision was made to accept the manuscript without peer review, a brief statement to this effect will instead be what is listed below.

Reviewer 1

In the attached manuscript the authors describe a new CRISPR/dCas9 (CRISPRa) approach for robust and specific gene regulation and provide a clear and comprehensive set of experiments highlighting a multimodal gene manipulation technology. This system provides the potential to rapidly investigate roles of targeted endogenous genes in diverse brain areas/cells.

Highlights of the work include detailed analysis of the CRISPRa as a gene activator of Bdnf and Fos, with a thorough in vitro analysis of outcomes. Not only do the authors show that Bdnf and Fos transcripts are elevated, they also confirm little to no off-target effects though a transcriptome wide analysis. Moreover, the manuscript provides new data on genes that are downstream of Bdnf in primary neuronal cultures. Although the general technology presented in this study is not novel, the authors clearly demonstrate a new utility for its application. CRISPRa has been available since 2015, and a manuscript reporting gene activation using Cas9 was published in 2017 (Liao et al.). However, this represents one of the more complete studies to date implementing CRISPRa, and one of the first studies utilizing CRISPRa in neurons.

Criticisms:

•It would be beneficial to add a bit more detail regarding screening and selecting specific sgRNA promoter sites in the main text. Although the notion of how site selection may have impacted variability of target activation was visited in the discussion, clearly this will have the most impact on gene regulation with application of the technology.

•Bdnf and Fos activation through CRISPRa are the focus of this paper. However, CRISPRa for Bdnf and Fos are often swapped throughout the paper. Consistency would be preferred throughout the manuscript, in particular for the experiments described in Figs 6 and 7.

•CRISPRi experiments were performed, but the data were not shown.

•It's a bit unclear from the MEA recording data how increased firing rates in the top 1/3 most active neurons, or increased spontaneous firing manifests from activating bdnf and/or fos transcripts. Perhaps unpacking these data a bit more with reference to previous studies, or providing further data/analysis would help bolster this section.

•The authors did an incredible amount of work however, it would've been great to see some functional application to this technology in vivo. The authors go through the trouble to surgically inject animals with virus, but do not mention any functional outcomes in vivo. Although I don't think this should impede the publication of the work in its current form, the authors would benefit greatly by adding some functional studies to this manuscript.

The manuscript clearly demonstrates the capabilities of CRISPRa in manipulating brain cells/tissue, and further provide a blueprint to quality control regarding applications for using CRISPR/Cas9 as an “activator” platform. I endorse publication, but some encouraged revisions would certainly increase the exposure and impact of the current work.

Reviewer 2

In the manuscript, the authors developed a neuron-optimized dual lentiviral CRISPR-based transcriptional activation (CRISPRa) system capable of gene induction and multiplexed gene regulation across several primary rodent neuron culture systems. They further demonstrated that this CRISPRa targeting two promoters in the multi-transcript gene Brain-derived neurotrophic factor (Bdnf) revealed specific transcriptional selectivity. They further demonstrated downstream transcriptional and physiological consequences of Bdnf regulation. Finally they illustrated that CRISPRa increased protein levels of a target gene in diverse brain structures in vivo. This molecular tool enables advances in our ability to control gene expression profiles in the brain. This CRISPRa system is interesting and the data provided sufficient support for its feasibility and usefulness. This article has been reviewed in Journal of Neuroscience before and the technical issues have been sufficiently addressed. Therefore this reviewer recommends its publication.
